# A phase I clinical study to evaluate rapid, high-volume, subcutaneous auto-injector tolerability with recombinant human hyaluronidase

**DOI:** 10.1007/s13346-025-01883-z

**Published:** 2025-05-30

**Authors:** David W. Kang, Robert J. Connor, Tara Nekoroski, Jo Ann M. Bitsura, Susan K. Kindig, Stephen P. Knowles, Michael J. LaBarre

**Affiliations:** 1 Halozyme Therapeutics, Inc. (Innovation Department), San Diego, CA USA; 2 Halozyme Therapeutics, Inc. (Bioanalytical Department), San Diego, CA USA; 3 Halozyme Therapeutics, Inc. (Clinical Department), San Diego, CA USA; 4 Formerly of Halozyme Therapeutics, Inc. (Clinical Department), San Diego, CA USA; 5 Halozyme Therapeutics, Inc, 12390 El Camino Real, San Diego, CA 92130 USA

**Keywords:** High-volume auto-injector, Recombinant human hyaluronidase PH20, Subcutaneous injection, Injection pain, Injection tolerability

## Abstract

Until recently, approved handheld auto-injectors (AIs) have been limited to volumes ≤ 2 mL. A prototype rapid high-volume AI (HVAI) that can deliver 10 mL in 30 s was developed to administer therapeutics co-formulated with a proprietary recombinant human hyaluronidase PH20 (rHuPH20). This phase I, open-label study assessed the tolerability of subcutaneous (SC) injections of 10% (100 mg/mL) immunoglobulin G (IgG) solution co-administered with 4000 U/mL rHuPH20, delivered using a syringe pump at a target rate of 5 or 10 mL/30 seconds or the prototype HVAI at a target rate of 10 mL/30 seconds in healthy human subjects. Subjects received 5 mL (Cohort A, *n* = 12) or 10 mL (Cohort B, *n* = 12) of test solution via syringe pump (injection visit 1), and 10 mL of test solution via HVAI (Cohorts A & B; injection visit 2). Primary endpoints were tolerability and safety outcomes. Secondary endpoints included HVAI injection duration. All 24 subjects completed visit 1; 23/24 completed visit 2. All injections were tolerated, with no serious adverse events (AEs). Following syringe pump administration, 6/24 subjects (25%) reported eight treatment-emergent AEs (TEAEs); after HVAI administration, 4/23 (17%) reported four TEAEs, all mild in severity. Mean (± SEM) injection duration via HVAI was 27.9 ± 0.8 s. Most subjects (91%, 21/23) experienced no or mild injection-site pain following HVAI administration, and 96% (22/23) said they would be willing to have the HVAI injection again. SC injection of a 10% IgG solution in combination with rHuPH20 was well tolerated at an injection rate of 10 mL/~30 s using the prototype HVAI.

**Supplementary Information:**

The online version contains supplementary material available at 10.1007/s13346-025-01883-z.

## Graphical abstract

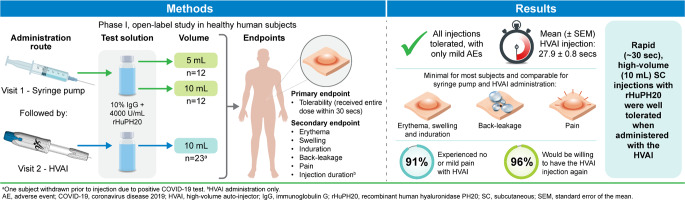

## Introduction

There has been steady growth in US Food and Drug Administration (FDA) approvals of subcutaneous (SC)-administered therapeutics [1]. Many products that had previously been administered intravenously are now being approved for SC delivery [2] and increasing numbers of therapeutics are being developed with SC delivery as the primary route of administration, without assessing intravenous (IV) administration [3].

SC delivery offers several advantages over IV infusion, including reduced risk of infection, infusion-related reactions, administration time, and healthcare costs [4–9]. These factors contribute to both patients and healthcare professionals often expressing a preference for SC over IV administration [4, 10, 11]. In addition, SC delivery allows for home-administration by a healthcare professional or self-administration of some biotherapeutics [4, 5].

However, SC administration has predominantly been restricted to volumes < 2 mL [12, 13] due to the limited capacity of SC tissue to accommodate larger volumes; larger volumes therefore require multiple injections or extended infusion times [14–16]. One established strategy for overcoming this volume limitation is coadministration of therapeutics with recombinant human hyaluronidase PH20 (rHuPH20). rHuPH20 is a highly purified form of the human hyaluronidase PH20 enzyme, which acts locally to degrade hyaluronan, temporarily eliminating the barrier to bulk fluid flow in the SC space [2, 17]. It thereby facilitates SC administration of co-administered therapeutics at larger volumes that would conventionally require IV administration [2, 14]. rHuPH20 has been approved by the US FDA and health regulatory authorities worldwide as an adjuvant to facilitate the dispersion and absorption of other injected drugs [18]. Nine biotherapeutics have been approved for SC administration with rHuPH20, either as coformulations [19–26] or for sequential administration [27].

Handheld auto-injectors (AIs) are injection devices containing enclosed prefilled syringes or cartridges, typically driven by a spring system [13]. These offer many advantages for patients and healthcare providers, including reduced patient anxiety since the needle is often obscured from view. Additionally, the risk of needlestick injuries, dosage errors, and accidental drug contamination are all reduced [28].

Until recently, AIs with rapid injection times were limited to low injection volumes; delivery of 1.0 mL in up to 15 s was considered the upper limit of delivery. A number of AIs delivering doses as high as 2.0 mL have been approved in the last decade, and development of higher volume (> 5.0 mL) AIs is ongoing [13]. However, many approved SC formulations for treating a range of different diseases, including cancer, autoimmune and central nervous system disorders, require administration of volumes of 5–23 mL per injection [3, 19–26]. By co-administering therapeutics with rHuPH20, development of rapid high-volume AIs (HVAIs) that can deliver volumes of 10 mL or even higher may become possible, potentially allowing some of these therapeutics that require higher volumes to be administered by AI in the future.

A prototype 10 mL HVAI for SC administration with rHuPH20 has been developed following extensive testing. A preclinical study in miniature pigs established baseline parameters of flow rate through a high-pressure syringe pump. This informed development of the prototype HVAI and demonstrated that the addition of rHuPH20 overcame volume and time constraints of SC administration in a dose-dependent manner across a range of concentrations of immunoglobulin (Ig) test solutions [17].

A subsequent study, also in miniature pigs, found that injection outcomes (including back-leakage, bleb size, swelling, and induration) were improved by the addition of rHuPH20 when administering an Ig solution using either a surrogate AI (syringe pump) or the prototype HVAI [29, 30]. An approximate 30-second injection duration was achieved with the HVAI, with minimal swelling, induration, and back-leakage [29, 30]; therefore, a 30-second rate of administration was planned for clinical evaluation.

Optimal injection parameters using the prototype HVAI were refined in further preclinical studies [30]. These studies in miniature pigs mimicked the parameters of the clinical evaluation reported here by using the same injection forces and injection duration, as well as the same clinically appropriate concentration of rHuPH20 (4000 U/mL) co-administered with a 10% immunoglobulin G (IgG) test solution [30]. An rHuPH20 concentration of 4000 U/mL was used to reduce the tissue back-pressure and allow rapid SC delivery; the rHuPH20 dose administered was less than the maximum rHuPH20 dose previously evaluated in clinical trials [31]. A 10% IgG solution was chosen as a representative biologic macromolecule test solution, based on FDA approval for the treatment of primary immunodeficiency (PI) via SC administration [32]. The same 10% IgG solution is also administered at much higher volumes (≥ 75 mL) and tolerated in patients with PI who self-administer IgG at home [33], thereby indicating that this solution would be suitable for use in this clinical evaluation.

This phase I clinical study sought to assess the tolerability of SC injections of a 10% IgG solution with rHuPH20, delivered at a target volume of 10 mL in ~ 30 s using a syringe pump or the prototype HVAI in healthy human subjects.

## Methods

### Study design

This was an open-label, multiple cohort phase I study in healthy human subjects.

#### Subjects

Inclusion criteria required subjects be 18 to 65 years of age; with intact, normal skin at the injection site, without obscuring tattoos, pigmentation, or lesions; without clinically significant abnormalities based on physical examination, clinical chemistry, hematology, and urinalysis; and with baseline general pain scores < 4 on the Numeric Rating Scale (NRS) [34], thereby excluding chronic pain conditions.

Key exclusion criteria included: a contraindication to Ig; a chronic pain condition or history of substance use disorder or drug abuse; pain at the abdominal injection site; and a known allergy to hyaluronidase or any component of the test solution. See supplementary material for full eligibility criteria.

#### Procedures

**Fig. 1:**
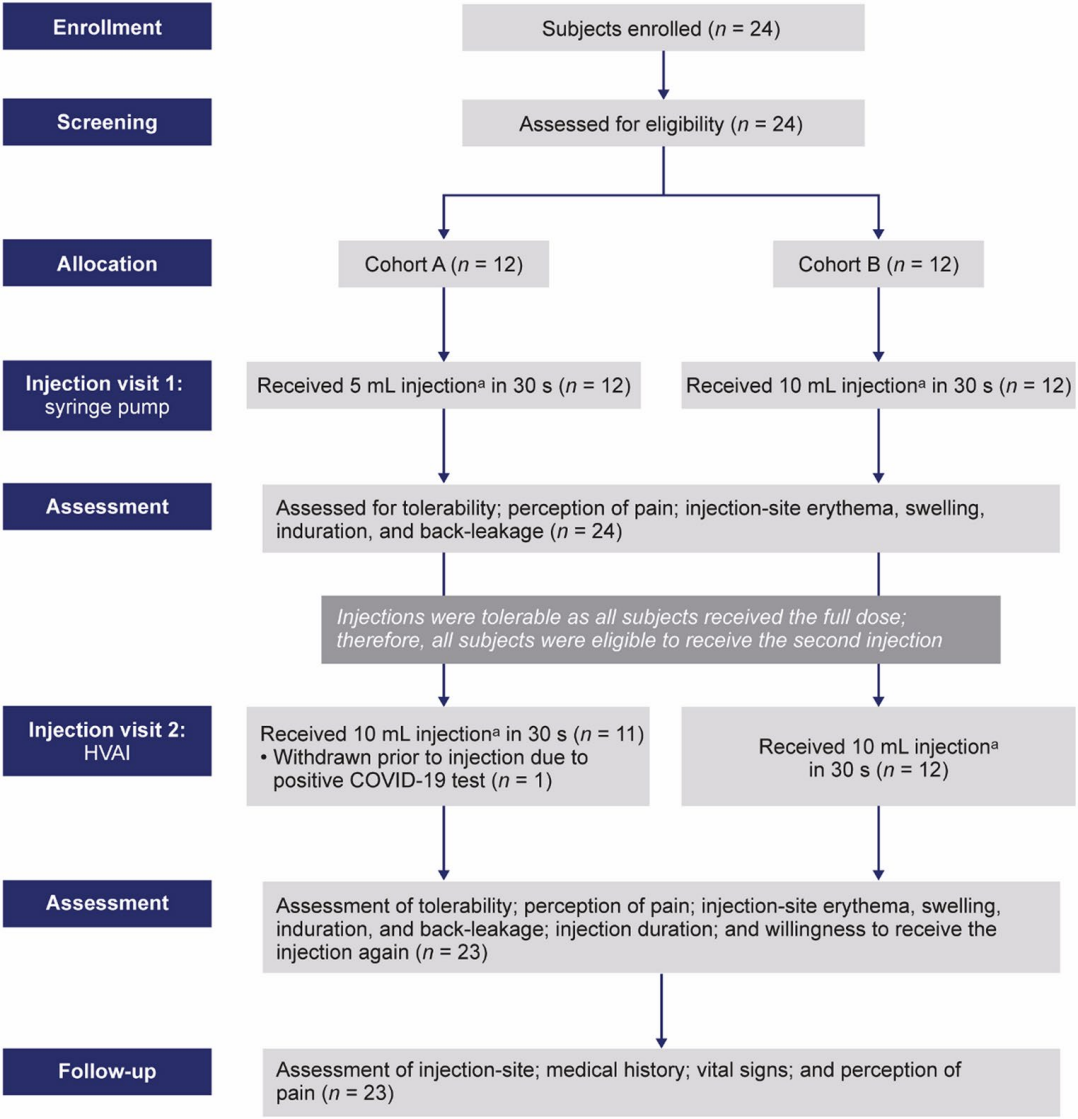
CONSORT flowchart. ^a^Test solution comprised 10% IgG with 4000 U/mL rHuPH20. COVID-19, coronavirus disease 2019; HVAI, high-volume auto-injector; IgG, immunoglobulin G; rHuPH20, recombinant human hyaluronidase PH20

A total of 24 subjects were assigned to two cohorts of equal size (*n* = 12/cohort). The study consisted of a screening and enrollment period, followed by two injection visits (Fig. 1) and safety follow-up. Injections took place over a total of 4 weeks (a single injection at each of the injection visits).

During injection visit 1, both cohorts (*n* = 24) were administered the test solution subcutaneously via syringe pump. First, subjects in Cohort A (*n* = 12) were administered 5 mL of the test solution in 30 s. If this volume was tolerated, Cohort B (*n* = 12) received injections at the higher volume of 10 mL in 30 s.

If Cohort B tolerated administration of 10 mL in 30 s via syringe pump during injection visit 1, both cohorts were administered 10 mL of test solution in 30 s via HVAI during injection visit 2.

Safety follow-up occurred for 8 weeks after a subject’s last injection, or until resolution of all adverse events (AEs) (whichever was longer) at 1, 4, and 8 weeks after the last injection.

#### Test solution

The test solution was composed of 10% (100 mg/mL) IgG solution (GAMMAGARD LIQUID [32], Baxalta Inc., a Takeda Company, Lexington, MA, USA) co-administered with 4000 U/mL rHuPH20. The viscosity of the IgG solution was 3.4 cP.

#### Syringe pump administration (injection visit 1)

Subjects received one SC injection of the test solution into the lower right abdominal quadrant using a Harvard PhD Ultra 4400 CP syringe pump (Harvard Apparatus, Holliston, MA, USA) [35]. The syringe pump was calibrated by the manufacturer prior to the study to ensure all injections would be delivered at the pre-programmed rate of 5 or 10 mL in 30 s. A 25-gauge (G) x 1-inch hypodermic thin-walled needle (Terumo Medical Corporation; Somerset, NJ, USA) connected to a 30-inch extension set (B. Braun Medical Ltd., Sheffield, UK) was used to deliver the test solution. The needle and extension set were routed through a surrogate AI handle that mimicked the HVAI and maintained needle depth from 7 to 12 mm.

#### HVAI administration (injection visit 2)

Subjects received one injection of the test solution into the lower left abdominal quadrant using the HVAI.

The HVAI is a proprietary device developed by Halozyme Therapeutics, Inc. It is single fixed-dose, spring-powered, disposable AI, designed to accommodate a 10 mL cyclic olefin copolymer syringe (SCHOTT, St Gallen, Switzerland). The device used a standard 25G × 1-inch needle (Becton Dickinson, Franklin Lakes, NJ, USA). It was designed to deliver the entire prefilled injection volume at an injection depth of approximately 10 mm.

### Assessments

#### Primary endpoints

The primary endpoint was injection tolerability, which was assessed at the syringe pump visit and HVAI visit. Tolerability was defined as the ability to receive the entire dose of test solution within the specified injection time (30 s) and without meeting stopping criteria (supplementary material). In addition, the subjects were asked to say ‘stop’ during the injection if they were unable to continue due to pain. If any subject reported an NRS pain score in the ‘severe’ (NRS 7–9) or ‘worst imaginable’ (NRS 10) categories, the trial was to be halted entirely.

Throughout injection administration, subjects were monitored for symptoms of injection-site reactions and allergic reactions or anaphylaxis. The incidence of subjects with TEAEs was evaluated by the investigator according to the Medical Dictionary of Regulatory Activities System Organ Class preferred term and for grade or severity, and relationship to study drug.

#### Secondary endpoints

The secondary endpoints for both injection visits were: the appearance and severity of erythema, swelling size, and induration (injection-site observations, all measured using a five-point modified Draize scoring scale); injection-site back-leakage; and subject perception of pain (measured using the NRS scoring scale). Pain was measured during needle insertion, but before injection; immediately after injection (0 min); and up to 360 min post-injection. The pain measurement immediately after injection was reported within seconds of the needle being withdrawn and was an assessment of the entire previous duration of the injection.

Methods for measuring pain must be considered carefully in a clinical evaluation such as this. Up to 11 pain scales are in common use, with NRS and the visual analog scale (VAS) the most commonly used for injection pain [1]. The NRS was chosen for this clinical evaluation because: the distinct categories of the NRS produce results that can be more easily analyzed statistically, reducing ambiguity in interpretation of the scores [36]; NRS allows for verbal scoring while VAS scoring requires subjects to make a physical mark on paper or electronically [36]; and with the NRS, patients report only on their perceived pain at the present time, reducing the likelihood of perceived pain being reported based on other pain experiences in recent times [37].

Additional secondary endpoints for the HVAI injection visit only were injection duration, which was recorded manually using a digital stopwatch (Thermo Fisher Scientific, Waltham, MA, USA), and subject responses (yes/no) to the patient-reported outcome (PRO) statement: “I would be willing to have this injection by the auto-injector again” (see supplementary methods).

### Statistical analysis

Standard descriptive summaries were carried out for each cohort’s demographic characteristics, tolerability, injection-site observations, back-leakage, NRS scores, injection duration with the HVAI, and response to the PRO question. Statistical comparisons of injection-site observations and back-leakage between Cohorts A and B within an injection visit, and between injection visits, were conducted using ordinary one-way ANOVA tests with Tukey’s multiple comparisons. All data analyses were performed using GraphPad Prism v10.3.1.

The intent-to-treat analysis set consisted of all subjects. This set was used for tolerability data summaries. The safety analysis set consisted of all subjects who received an injection of any amount of test solution. This set was used for the safety endpoint parameters, safety data summaries, baseline characteristic summaries, and selected tolerability data summaries.

## Results

### Demographics and baseline characteristics

The study enrolled 24 healthy subjects (Table 1) with a mean age of 45 years (range = 19–62 years) and a mean body mass index of 30 kg/m^2^ (range = 22–46 kg/m^2^). Most individuals were white (79%), and more than half of the subjects were female (63%).

**Table 1:** Demographic and baseline characteristics

	Cohort A (*N* = 12)	Cohort B (*N* = 12)
**Age, years**		
Mean (SD)	44.8 (11.73)	44.5 (14.93)
Median (min, max)	42.0 (24, 61)	48.0 (19, 62)
**Sex**,** n (%)**		
Male	6 (50.0)	3 (25.0)
Female	6 (50.0)	9 (75.0)
**Race**,** n (%)**		
American Indian or Alaska Native	0	0
Asian	0	0
Black or African American	2 (16.7)	2 (16.7)
Native Hawaiian or other Pacific Islander	1 (8.3)	0
White	9 (75.0)	10 (83.3)
Other	0	0
**Ethnicity**,** n (%)**		
Hispanic or Latino	7 (58.3)	8 (66.7)
Not Hispanic or Latino	5 (41.7)	4 (33.3)
**Weight**,** kg**		
Mean (SD)	83.85 (12.835)	86.22 (18.637)
Median (min, max)	81.90 (62.6, 105.0)	87.10 (66.0, 128.9)
**Height**,** cm**		
Mean (SD)	172.07 (9.153)	164.22 (8.185)
Median (min, max)	175.50 (160.3, 185.7)	161.25 (155.4, 179.3)
**BMI**,** kg/m**^2^		
Mean (SD)	28.45 (4.832)	31.84 (5.804)
Median (min, max)	27.40 (21.7, 39.1)	30.00 (26.2, 45.7)

BMI, body mass index; max, maximum; min, minimum; SD, standard deviation

Subjects were divided equally between Cohort A (*n* = 12) and Cohort B (*n* = 12). No subjects were discontinued early, although 1 subject in Cohort A did not receive the second of the two planned injections due to an AE of coronavirus disease 2019 infection. The subject did not attend follow-up visit 1 due to the illness, but did attend follow-up visits 2 and 3 and therefore was considered to have completed the study.

### Safety

All 24 subjects completed the study and were included in the safety analysis (Table 2). No serious AEs (SAEs) were reported, and no subjects required analgesics. There were no deaths or discontinuations due to AEs. Overall, 8 of 24 subjects (33%) reported 12 TEAEs, all of which were mild in severity, and of which 6 TEAEs (25%) were considered to be definitely related to study drug.

**Table 2:** Summary of adverse events

	Syringe pump injection visit (injection visit 1)		HVAI injection visit (injection visit 2)		
	Cohort A 5 mL/ 30 s (*N* = 12)	Cohort B 10 mL/ 30 s (*N* = 12)	Cohort A 10 mL/ 30 s (*N* = 11)	Cohort B 10 mL/ 30 s (*N* = 12)	Overall (*N* = 24)
**Total number of TEAEs**	6	2	4	0	12
**Subjects with at least 1 TEAE, n (%)**	4 (33.3)	2 (16.7)	4 (36.4)	0	10 (41.7)
Mild	4 (33.3)	2 (16.7)	4 (36.4)	0	10 (41.7)
Moderate	0	0	0	0	0
Severe	0	0	0	0	0
**TEAEs by system/organ class**,** n (%)**					
Ear and labyrinth disorders: ear congestion	1 (8.3)	0	0	0	1 (4.2)
General disorders and administration site conditions	0	2 (16.7)	2 (18.2)	0	4 (16.7)
Injection-site pruritus	0	1 (8.3)	1 (9.1)	0	2 (8.3)
Injection-site rash	0	0	1 (9.1)	0	1 (4.2)
Non-cardiac chest pain	0	1 (8.3)	0	0	1 (4.2)
Infections and infestations: COVID-19	1 (8.3)	0	1 (9.1)	0	2 (8.3)
Skin and subcutaneous tissue disorders: pruritus	4 (33.3)	0	1 (9.1)	0	5 (20.8)
**TEAES by relationship to study drug**,** n (%)**					
Definitely related	2 (16.7)	1 (8.3)	3 (27.30)	0	6 (25.0)
Probably related	2 (16.7)	0	0	0	2 (8.3)
Possibly related	0	1 (8.3)	0	0	1 (4.2)
Unlikely related	0	0	0	0	0
Not related	0	0	1 (9.1)	0	1 (4.2)
**SAEs**					
Total number of SAEs	0	0	0	0	0
Subjects requiring analgesia, n (%)	0	0	0	0	0
Subjects with AEs leading to discontinuation, n (%)	1 (8.3)	0	0	0	1 (4.2)
Deaths, n (%)	0	0	0	0	0

Subjects were counted only once under the category of their most drug-related event and most severe event, respectively. Subjects were counted only once within each system organ class and preferred term

AE, adverse event; COVID-19, coronavirus disease 2019; HVAI, high-volume auto-injector; SAE, serious adverse event; TEAE, treatment-emergent adverse event

#### AEs following syringe pump administration

Following syringe pump administration (injection visit 1), 8 TEAEs were reported in 6 subjects. All were mild in severity and 3 were considered to be definitely related to the study drug. These were pruritus (Cohort A: 2/12 subjects, 17%) and injection-site pruritus (Cohort B: 1/12 subjects, 8%). Additionally, there were 2 reported cases of pruritus that were considered to be probably related to the study drug (Cohort A: 2/12 subjects, 17%). One reported TEAE of non-cardiac chest pain was considered possibly related to the study drug (Cohort B: 1/12 subjects, 8%).

#### AEs following HVAI administration

Following HVAI administration (injection visit 2), four TEAEs were reported in 4 subjects. All were mild in severity. Three were considered to be definitely related to the study drug (pruritus, injection-site pruritus, and injection-site rash, each in 1/11 [9%] subjects in Cohort A) and one was considered not related to the study drug.

#### Tolerability

All injections were tolerated by all subjects across both cohorts, when administered via either syringe pump or the HVAI.

#### Tolerability of syringe pump administration

Since all injections were tolerated in Cohort A (5 mL received in 30 s, *n* = 12), this permitted subjects in Cohort B (*n* = 12) to receive the higher volume of 10 mL of test solution in 30 s. All injections in Cohort B were also completed and were tolerable, and so all subjects were eligible to receive HVAI injections during the second visit.

#### Tolerability of HVAI administration and injection duration

Subjects who were administered injections in Cohorts A (*n* = 11) and B (*n* = 12), received the full dose (10 mL) within 30 s via HVAI without meeting any stopping criteria; therefore, all doses were tolerable.

**Fig. 2:**
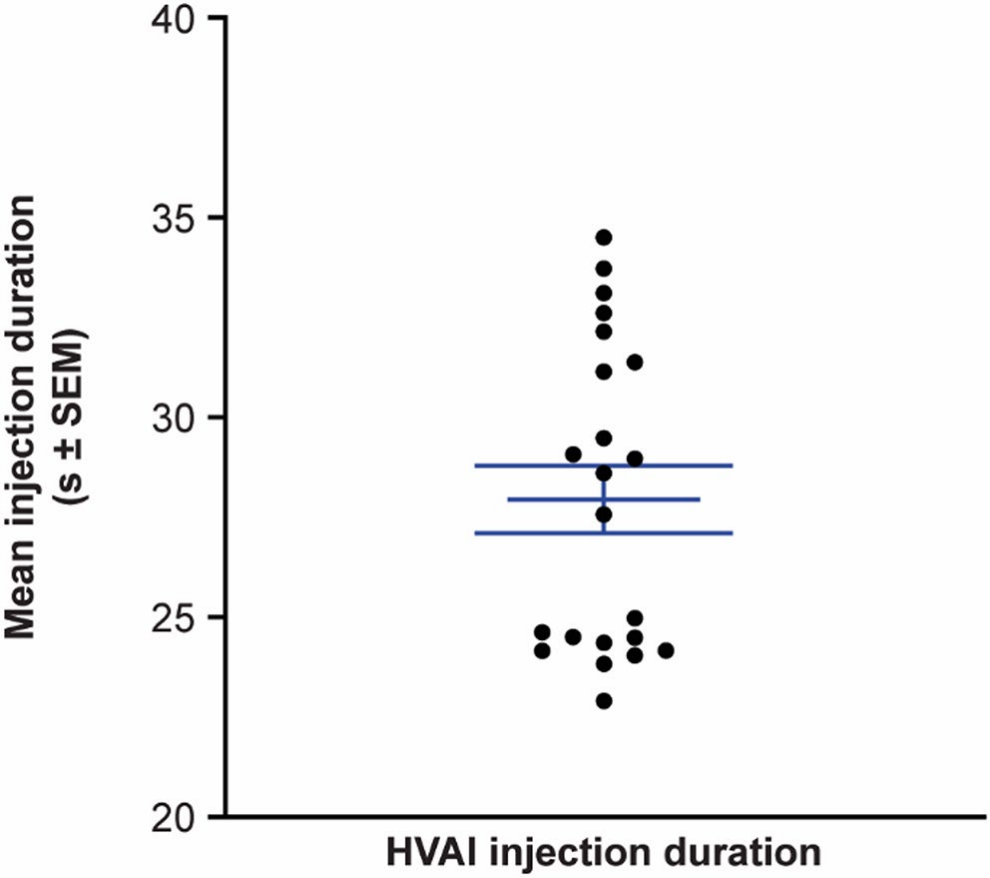
Mean (± SEM) injection duration following HVAI administration of 10 mL of test solution^a^ across both cohorts. Dots represent injection duration for all 22 subjects for which injection duration was recorded. A timing error occurred for the remaining 1 subject who received the second injection; therefore, injection duration was not recorded. ^a^Test solution comprised 10% IgG with 4000 U/mL rHuPH20. HVAI, high-volume auto-injector; IgG, immunoglobulin G; rHuPH20, recombinant human hyaluronidase PH20; SEM, standard error of the mean

Mean (± standard error of the mean) injection duration of the test solution administered via HVAI across both cohorts was 27.9 ± 0.8 s (Fig. 2). All injections were completed between 22.9 and 34.5 s.

### Post-injection erythema, swelling, and induration

#### Erythema, swelling, and induration following syringe pump administration

**Fig. 3:**
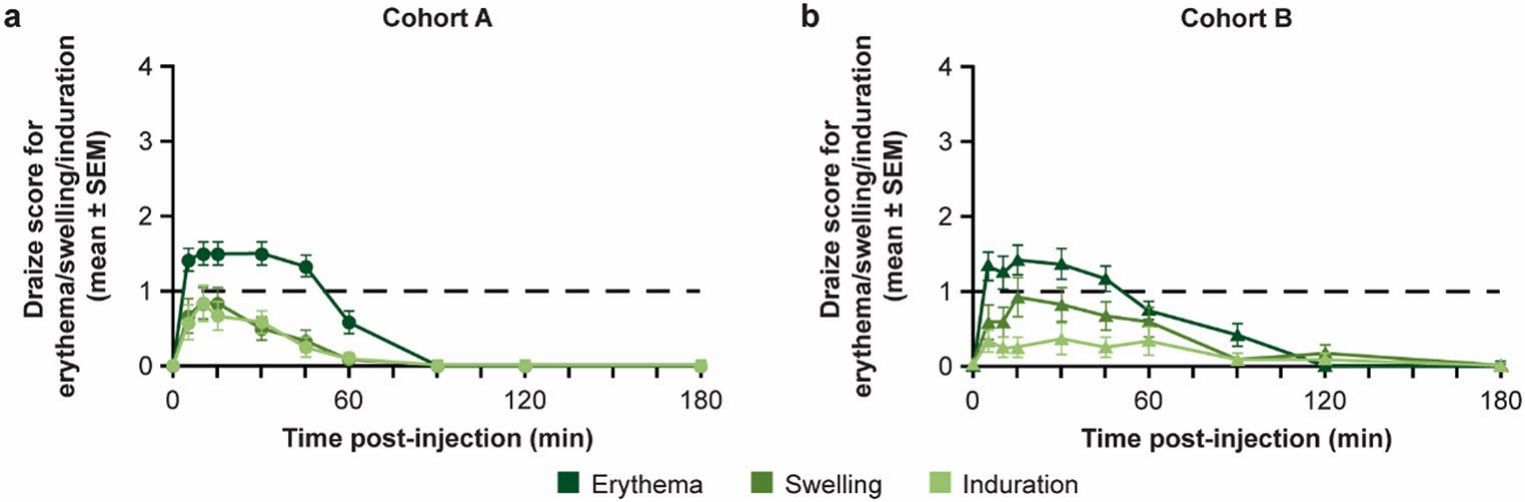
Mean (± SEM) Draize scores for erythema, swelling, and induration following **a**) syringe pump administration of 5 mL test solution^a^ in Cohort A and **b**) syringe pump administration of 10 mL test solution^a^ in Cohort B. The dotted line represents a minimal or ‘barely perceptible’ Draize score. ^a^Test solution comprised 10% IgG with 4000 U/mL rHuPH20. IgG, immunoglobulin G; rHuPH20, recombinant human hyaluronidase PH20; SEM, standard error of the mean

Erythema, swelling, and induration were comparable across both cohorts (Fig. 3), with scores increasing immediately post-injection, peaking at 10 min for Cohort A, and at 15 min (erythema and swelling) and 30 min (induration) for Cohort B. Scores then decreased steadily up to 60 min post-injection, with complete resolution by 180 min for both cohorts. The differences between Cohort A (5 mL) and Cohort B (10 mL) in erythema, swelling, and induration at 60 min post-injection were minimal and insignificant (*P* > 0.99, *P* = 0.38, *P* = 0.44, respectively).

#### Erythema, swelling, and induration following HVAI administration

**Fig. 4:**
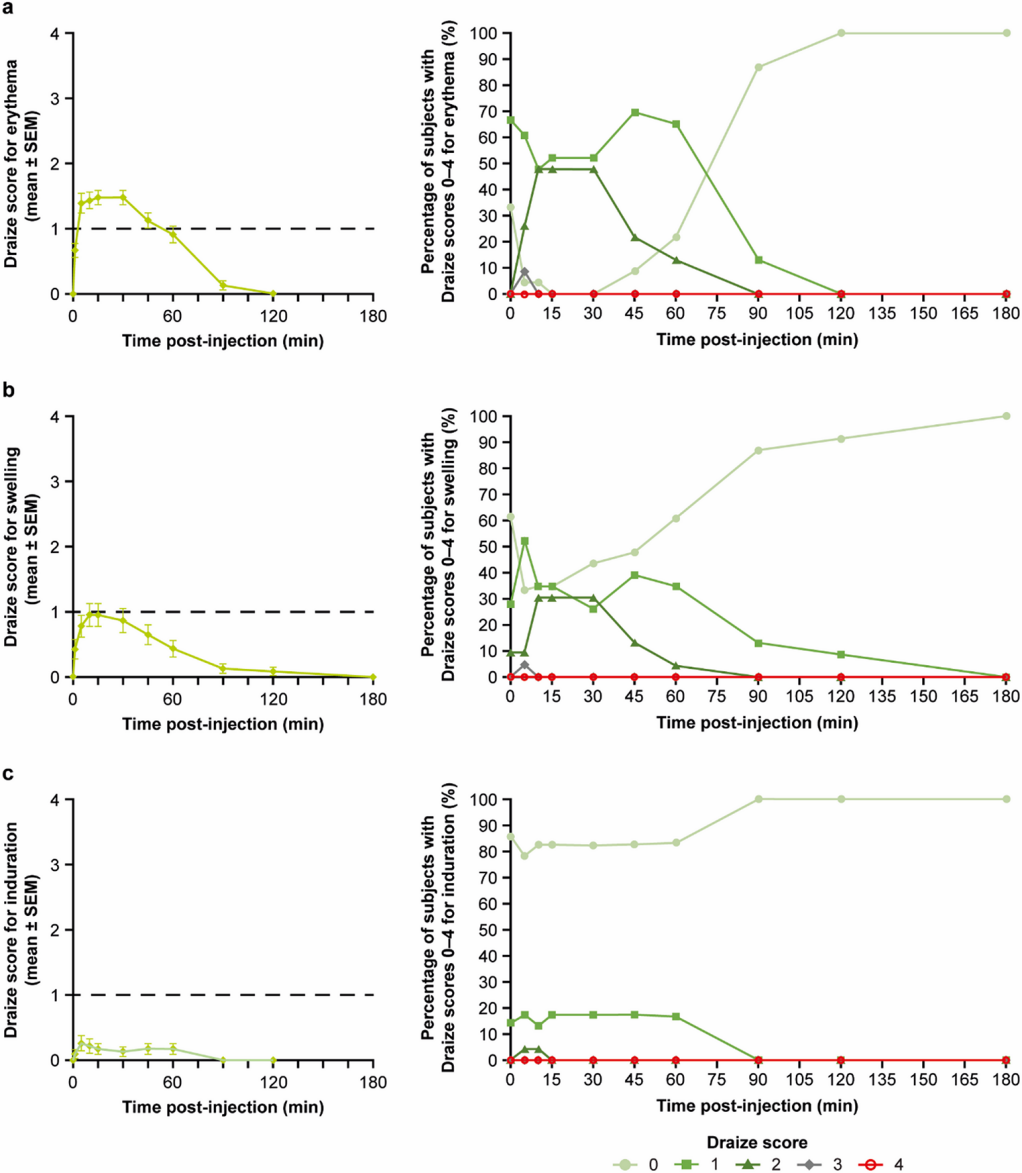
Mean (± SEM) Draize scores and breakdown of Draize scores as a percentage of subjects for erythema (**a**), swelling (**b**), and induration (**c**) following HVAI administration of 10 mL test solution^a^ across both Cohort A and Cohort B^b^. Mean scores for erythema, swelling, and induration are shown on the left. Breakdown of Draize scores as a percentage of subjects is shown on the right. The dotted line represents a minimal or ‘barely perceptible’ Draize score. ^a^Test solution comprised 10% IgG with 4000 U/mL rHuPH20. ^b^There was no difference in experimental conditions between Cohorts A and B for HVAI administration. Both cohorts received 10 mL of test solution via HVAI. HVAI, high-volume auto-injector; IgG, immunoglobulin G; rHuPH20, recombinant human hyaluronidase PH20; SEM, standard error of the mean

Erythema, swelling, and induration were minimal for all subjects (Fig. 4), peaking at 15, 10, and 5 min respectively across both cohorts, with complete resolution by 180 min.

There were no differences in erythema, swelling, and induration (*P* = 0.83, *P* > 0.99, *P* = 0.91 at 60 min, respectively) between syringe pump administration of 10 mL (Cohort B) and HVAI administration of 10 mL (Cohorts A and B combined).

### Injection-site back-leakage

Mean injection-site back-leakage volume was low and proportional to the injection volume following syringe pump administration (Cohort A, 0.005 mL [0.10%]; Cohort B, 0.011 mL [0.11%]) and following HVAI administration (Cohort A and B combined, 0.009 mL [0.09%]). Back-leakage volumes following HVAI administration (10 mL) were comparable with the back-leakage measured for Cohort B (10 mL) with the syringe pump (*P* = 0.99).

See supplementary material for details.

### Injection-site pain

#### Injection-site pain following syringe pump administration

At needle placement, but before the injection began, the majority of subjects (19/24, 79%) reported no pain (NRS 0), and all other subjects reported mild pain (5/24, 21%). No subjects reported pain greater than mild pain (NRS > 3).

**Fig. 5:**
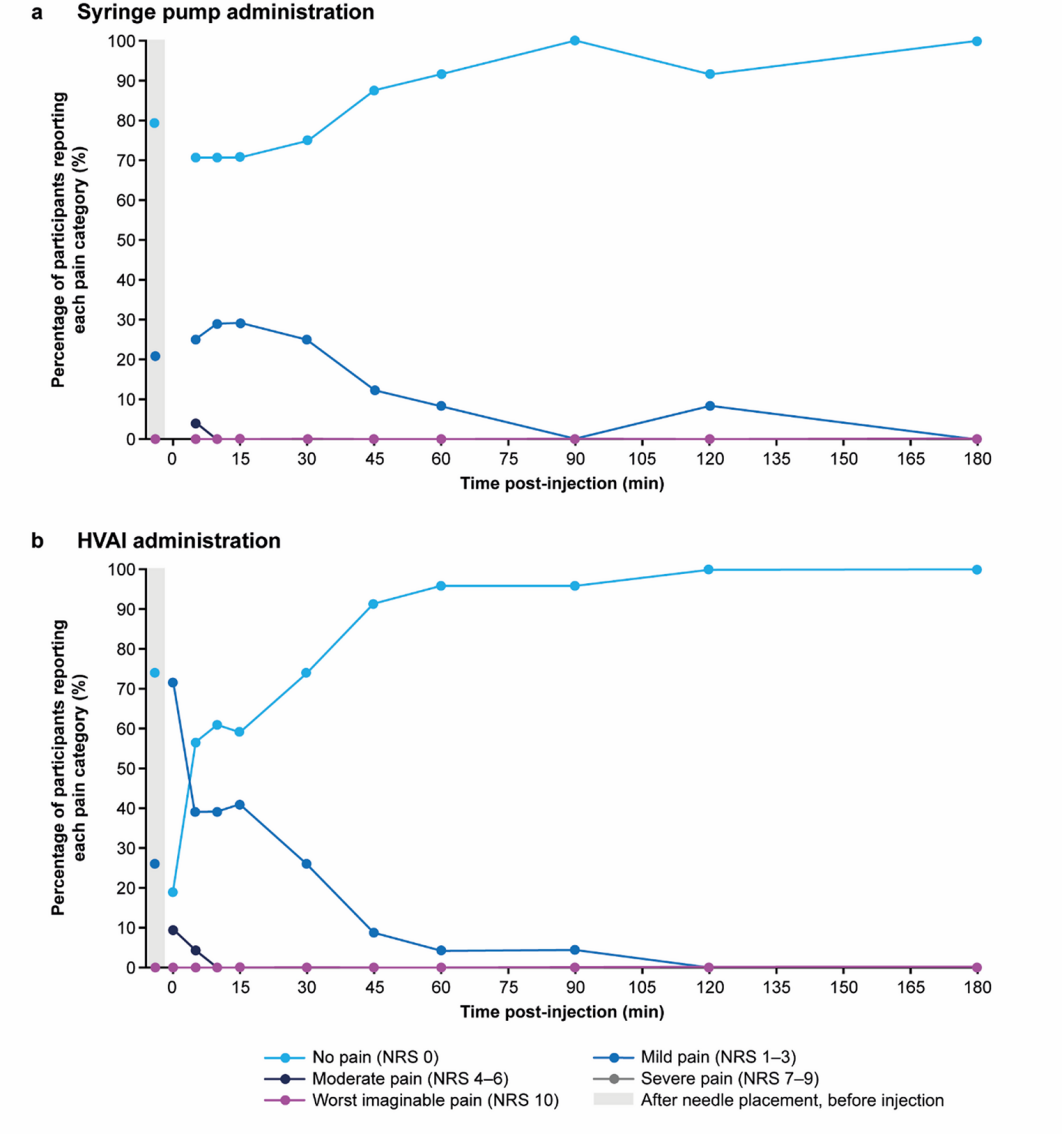
Injection-site pain according to the NRS (0–10 scale) following (**a**) syringe pump administration^a^ of 5 or 10 mL test solution^b^ and (**b**) HVAI administration^c^ of 10 mL test solution^b^ as a percentage of subjects reporting each pain category at each timepoint. ^a^No data were collected immediately after injection (0 min) with the syringe pump (panel a). ^b^Test solution comprised 10% IgG with 4000 U/mL rHuPH20. ^c^Scores were recorded for 21 subjects at 0 min post-injection and for 22 subjects at 15 min post-injection due to occasions where the investigator forgot to ask the subject or did not note their score in the report. HVAI, high-volume auto-injector; IgG, immunoglobulin G; NRS, Numeric Rating Scale; rHuPH20, recombinant human hyaluronidase PH20

The majority of subjects (23/24, 96%) reported no pain or mild pain at 5 min post-injection, with rapid resolution observed over time (Fig. 5a). In Cohort A, 1 subject (1/24, 4%) reported moderate pain (NRS 4) at 5 min after injection and by 10 min post-injection, 100% of subjects (24/24) scored no pain or mild pain. No subject reported pain higher than moderate pain during the post-injection and follow-up period.

#### Injection-site pain following HVAI administration

Due to a recording error, scores were not recorded for 2 subjects at 0 min post-injection, and 1 subject at 15 min post-injection.

At needle placement, but before the injection began, no subjects reported pain greater than mild pain (NRS 1–3), and the majority (17/23, 74%) reported no pain (NRS 0). Most subjects (19/21, 90%) reported no pain (NRS 0) or mild pain (NRS 1–3) immediately post-injection (0 min), increasing to 96% (22/23) by 5 min post-injection and 100% (23/23) by 10 min post-injection that subjects reported no pain (Fig. 5b). At 0 min, 2 subjects (2/21, 10%) reported moderate pain (NRS 6). At 5 min post-injection, 1 subject (1/23, 4%) reported moderate pain (NRS 4). Overall, 91% (21/23) of subjects reported no pain or only mild pain as their highest pain category following HVAI injection. There was no trend observed between injection-site pain and injection duration.

#### Subjects reported willingness to repeat HVAI injection

**Fig. 6:**
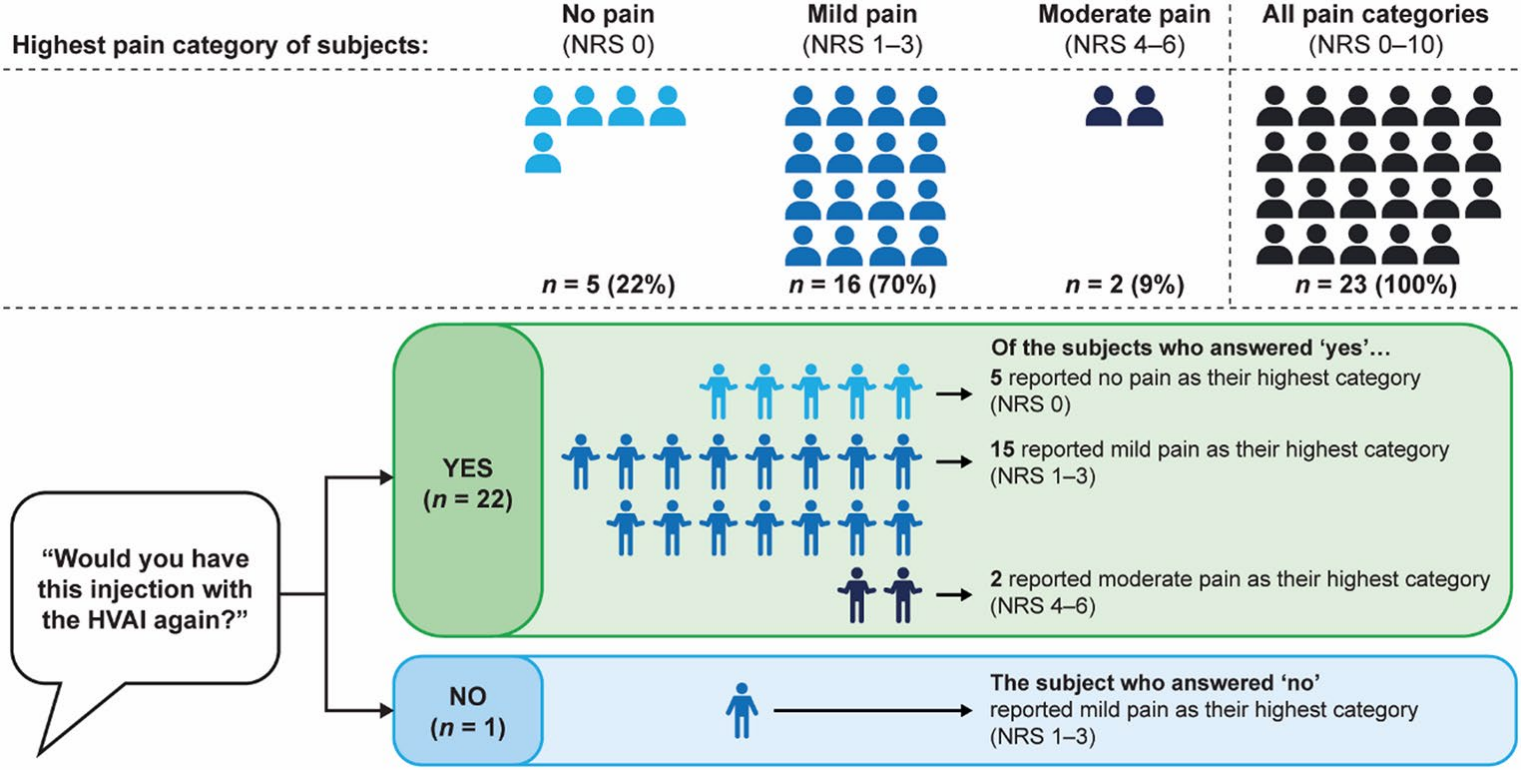
NRS scores and PROs following HVAI administration of 10 mL test solution^a^ and a breakdown of NRS scores reported by each subject across both cohorts. ^a^Test solution comprised 10% IgG with 4000 U/mL rHuPH20. HVAI, high-volume auto-injector; IgG, immunoglobulin G; NRS, Numeric Rating Scale; PRO, patient-reported outcome; rHuPH20, recombinant human hyaluronidase PH20

After receiving the HVAI injection, 96% (22/23) of subjects indicated that they would be willing to have the HVAI injection again (Fig. 6).

The highest reported pain category was no pain (NRS 0) or mild pain (NRS 1–3) for 21/23 subjects (91% total; no pain, 5/23 [22%]; mild pain, 16/23 [70%]). The only participant in the study who indicated that they would not be willing to have the injection again with the HVAI reported mild pain as their highest pain category. Both subjects who reported their highest pain category as moderate reported an NRS score of six, and both responded that they would be willing to have the injection again with the HVAI.

## Discussion

### Summary of key findings

rHuPH20 reduces the barrier to bulk fluid flow in the SC space, allowing for greater dispersion and absorption of co-administered therapeutics, and thereby facilitating SC injection of large volumes [38]. This study was designed to assess the tolerability of rapid, high-volume injections co-administered with rHuPH20 using the prototype HVAI.

This clinical evaluation showed that rapid (30 s) SC injection of 10 mL of a 10% solution of IgG in combination with rHuPH20 was well tolerated by all subjects using both a syringe pump and the prototype HVAI. A 25G needle was used to achieve fast flow rates. All TEAEs were mild in severity and there were no SAEs or requirement for analgesics with either administration route. SC injection of 10 mL in 30 s may be tolerable for other therapeutics co-administered with rHuPH20 using the HVAI, and this volume and injection duration are appropriate parameters for further clinical evaluation.

Scores for erythema, swelling, and induration, and volume of back-leakage were low and comparable for syringe pump and HVAI administration. These findings are consistent with the experience of patients dosed subcutaneously with commercial biotherapeutics containing rHuPH20 through infusion sets. For example, injection-site reactions from commercial products co-formulated with rHuPH20 are usually mild or moderate and resolve [19–26].

High-volume, high-flow rate SC administration with rHuPH20 is generally well tolerated, and patients often prefer high-volume SC injections facilitated with rHuPH20 over traditional IV infusion [10, 39]. Despite the high volume, rapid injection times, and wider gauge needle (25G) of the HVAI used in the current study, the majority of subjects experienced only mild or no pain at the injection site, both at needle insertion before injection (74%, 17/23 subjects), and immediately after injection (91%, 21/23 subjects). The 2 subjects who experienced moderate pain after injection responded ‘yes’ to the PRO question, “I would be willing to have this injection by the auto-injector again”.

The factors influencing injection-site pain are complex. A recent review of SC administration of volumes > 1 mL shows a limited impact of injection volume on pain in some studies [13], while others suggest that pain increases with volume [13, 40–43]. For example, in a study in which large volumes (> 5 mL) of a 20 cP solution without rHuPH20 were administered abdominally via syringe pump, data trends suggested that 10 mL injections were more painful than 5 mL volumes [35]. With volumes > 2 mL, more rapid injection may also result in greater pain. In a previous clinical study without rHuPH20, 3.5 mL injections administered SC in 1 min were considered more painful than the same volume of solution administered SC over 4–10 min [44]. However, the relationship between injection rate and pain remains uncertain, as other studies suggest no or limited impact of injection rate on pain (reviewed in [13]). Finally, our results with a 25G needle corroborate findings from a clinical study with a wider gauge (24G) needle that resulted only in mild to no pain upon needle insertion [45]. This suggests that, even at relatively wide needle gauges, pain can still be mild. The low reported pain levels in this study, even at the large volume and rapid injection time that may be expected to increase pain for some injections, could be due to the formulation of the test solution with rHuPH20, which decreases tissue pressure and force required for injection [17, 38].

### Clinical implications

Rapid SC administration of a high volume (10 mL) of a concentrated biologic (10% IgG) co-formulated with rHuPH20 in ~ 30 s using an HVAI is tolerable for patients. It is probable that the presence of rHuPH20 contributed to such large volumes being tolerated; however, no direct comparison without rHuPH20 was tested. It was agreed between the clinical trial sponsor and the regulatory agency that such rapid high-volume SC injections would not be administered without hyaluronidase to human subjects for reasons of safety. This decision was informed by results from preclinical studies in miniature pigs, in which injections without rHuPH20 resulted in larger blebs and more severe and persistent swelling and induration at the injection site in comparison with injections with rHuPH20 [30].

The 10-mL volume administered in this study is up to 5 times the typical expected SC volume limit for an AI [13]. These results are expected to be broadly applicable to enable SC delivery of common biotherapeutics and non-biologic therapeutics that require SC dosing volumes in excess of 2 mL. The tolerability and short duration of the injection are consistent with patient preference and human factor studies that suggest 30 s is a suitable duration for self-administration [46]. Importantly, the majority of injections with the HVAI were accompanied by no to mild pain. Injection-site pain contributes to a suboptimal patient experience when using SC AIs, with perceived or actual pain being shown to increase anxiety, reduce adherence, and reduce patient confidence in their ability to self-administer injections correctly [47]. Therefore, it is anticipated that use of the HVAI may minimize occurrence of these issues.

The ability to administer larger volumes of therapeutics rapidly with an HVAI has the potential to expand the number of therapies that could be administered in a home setting, with the associated benefits to patients and healthcare providers of reduced treatment burden, improved quality of life, and reduced treatment costs [4–9].

### Strengths and limitations

This study has a number of limitations. Injections were administered to a relatively small group of subjects (*n* = 24 for the syringe pump visit and *n* = 23 for the HVAI visit) and data on subjects with comorbidities should be evaluated in future studies. Additionally, there may be differences in how pain is perceived by patients who regularly receive treatment versus subjects who do not. Further research on the intricacies of pain perception in patients who regularly receive treatment is required, as pain can be multifaceted and influenced by various factors such as environment and patient expectations [36].

### Future research and next steps

The upper volume limit of rHuPH20-mediated SC injections has not yet been determined. This study demonstrated that rapid HVAI injection of 10 mL of a therapeutic was tolerable; however, as this volume is not sufficient to meet the volume requirements of all approved SC-administered therapeutics, future studies may evaluate larger volumes. Similarly, future studies may investigate whether shorter injection durations are feasible and evaluate the effect of needle gauge on tolerability.

It may also be beneficial to further investigate the role of rHuPH20 concentration in the tolerability of HVAI injections. This study utilized an rHuPH20 concentration which was 2× higher (4000 U/mL) than in already approved rHuPH20 co-formulated products (2000 U/mL) that are delivered at slower flow rates (ranging from 1 mL/min to 11.2 mL/min in comparison with the 20 mL/min of this study) [19–26]. This concentration was well tolerated when used with the HVAI. However, additional clinical assessment of other concentrations and delivery rates may be desirable.

## Conclusion

SC injection of a 10% solution of Ig in combination with rHuPH20 was well tolerated in human subjects at an injection volume and rate of 10 mL when administered in approximately 30 s using a prototype HVAI. The majority of subjects (91%, 21/23) experienced no or mild injection-site pain following administration of the test solution when using the prototype HVAI, and almost all subjects (96%: 22/23) responded that they would be willing to have such an injection again.

rHuPH20 currently facilitates rapid SC administration in nine approved products [19–27]. In this study, the presence of rHuPH20 is believed to have facilitated rapid (< 30 s) SC injection of concentrated biologics at high volumes (10 mL), thus contributing to effective HVAI development. This may also deliver benefits associated with AIs — which include reduced injection time, increased patient compliance, and the possibility of at home- or self-administration — to patients across a greater range of diseases.

## Electronic supplementary material

Below is the link to the electronic supplementary material.

Supplementary Material 1

## Data Availability

Halozyme Therapeutics, Inc. follows policies established by the International Committee of Medical Journal Editors. The studies were conducted by Halozyme Therapeutics, Inc., and the data are held by the company. Additional information about the studies and/or datasets can be obtained by contacting Halozyme Therapeutics, Inc.: 12390 El Camino Real, San Diego, CA 92130, USA; Phone: +1.858.794.8889; Email: publications@halozyme.com.
