# Supplementary material for: A phase I clinical study to evaluate rapid, high-volume, subcutaneous auto-injector tolerability with recombinant human hyaluronidase
Source: Drug Deliv Transl Res. 2025 May 30;16(1):95–107. doi: 10.1007/s13346-025-01883-z (PMC12682915; doi:10.1007/s13346-025-01883-z)
Supplement: Supplementary file 1 — Supplementary Material 1 [file 13346_2025_1883_MOESM1_ESM.docx]

A phase I clinical study to evaluate rapid, high-volume, subcutaneous auto-injector tolerability with recombinant human hyaluronidase

David W. Kang,^1^ Robert J. Connor,^1^ Tara Nekoroski,^2^ Jo Ann M. Bitsura,^3^ Susan K. Kindig,^4^ Stephen P. Knowles,^4^ Michael J. LaBarre,^1^

*^1^Halozyme Therapeutics, Inc. (Innovation Department), San Diego, CA, USA*

*^2^Halozyme Therapeutics, Inc. (Bioanalytical Department), San Diego, CA, USA*

*^3^Halozyme Therapeutics, Inc. (Clinical Department), San Diego, CA, USA*

*^4^Formerly of Halozyme Therapeutics, Inc. (Clinical Department), San Diego, CA, USA*

**Corresponding author:** David W. Kang

Email: publications@halozyme.com

Halozyme Therapeutics, Inc., San Diego, CA, USA

12390 El Camino Real, San Diego, CA 92130, USA

publications@halozyme.com; +1-858-794-8889

**Supplementary material**

## Supplementary methods

### Full eligibility criteria

Inclusion criteria required subjects to be 18 to 65 years of age; provide a signed consent form; have intact, normal skin at the injection site without any obscuring tattoos, pigmentation, or lesions; present with vital signs within the normal range or vital-sign abnormalities assessed as not clinically significant by the investigator; present clinical chemistry, hematology, and urinalysis within the normal range or assessed as not clinically significant within the 14 days prior to administration; provide a negative urine pregnancy test prior to the first and second injection visits (female subjects); present a negative toxicology screen test; have adequate venous access in at least one upper extremity; provide a negative nasal coronavirus disease 2019 test swab; and provide baseline general pain scores < 4 on a Numeric Rating Scale (NRS; 0–10 scale with scores corresponding to 0 [no pain], 1–3 [mild pain], 4–6 [moderate pain], 7–9 [severe pain], and 10 [worst imaginable pain]), ruling out chronic pain conditions.

Exclusion criteria were: having contraindication to immunoglobulin; showing clinical laboratory evidence of renal insufficiency or failure; having volume depletion; contracting sepsis; having paraproteinemia; having diabetes mellitus; receiving known nephrotoxic drugs; having a chronic pain condition or history of substance use disorder or drug abuse; presenting with pain at the abdominal injection site; being pregnant, planning to become pregnant, or being a breastfeeding female subject; being unwilling to use highly effective birth control throughout the study, being of reproductive potential and not accepting potential risks to current or future fertility; having a known allergy to hyaluronidase or any components of the test solution; being at high risk for thrombosis or hemolysis; having taken anticoagulants or analgesics within 12 hours prior to injections; having a known clinically significant, intercurrent illness or major systemic disease that would unduly risk the subject’s safety or interfere with completion of the study and interpretation of results; or participating in a study of any investigational drug or device within 30 days or five half-lives prior to enrollment, whichever was longer.

### Stopping criteria for injections

Injections were not considered tolerated if any of the following criteria were met:

1. At least 2 subjects had a Grade 2 (per Common Terminology Criteria for Adverse Events Version [CTCAE v] v5) allergic reaction or injection-site reaction
2. Any subject in a cohort had: a Grade 3 or higher (per CTCAE v5) allergic reaction or injection-site reaction; any Grade 4 or higher adverse event (regardless of attribution to treatment); any signs or symptoms of thrombosis, hemolysis, or acute kidney injury

### Secondary endpoint assessment

Erythema, swelling, and induration were assessed by the investigator using a 5-point modified Draize scoring scale: on the grading scale, 0 = no reaction, 1 = very slight reaction, 2 = slight reaction, 3 = moderate reaction, and 4 = severe reaction. Scores were collected at 5, 10, 15, 30, 45, 60, and 90 minutes post-injection, followed by hourly score collection up to 6 hours post-injection.

Back-leakage of fluid was collected by dabbing a pre-weighed wicking spear at the injection site immediately post-injection for 30 seconds, which was quantified by weight. Back-leakage was calculated from the weight difference of the wicking spear before and after collecting the leaked fluid, which was converted to volume of back-leakage (1 mg is approximately 1 μL).

Pain at the injection site was self-assessed by the subject on a 0–10 NRS, on which 0 represented no pain, and 10 represented the worst imaginable pain [1]. NRS scores were compared immediately after needle insertion (prior to the start of injection) versus the NRS score immediately after injection of the test solution, and at 5, 10, 15, 30, 45, 60, 90, and 120 minutes post-injection, followed by hourly score collection up to 6 hours post-injection. This was also analyzed by calculating the proportion of subjects who required analgesia for treatment of pain following each injection, and the time to resolution of pain.

## Supplementary results

### Post-injection erythema, swelling, and induration

#### Syringe pump administration

In Cohort A (5 mL), mean ± standard error of the mean (SEM) Draize scores for erythema, swelling, and induration peaked at 10 minutes, with scores of 1.5 ± 0.2, 0.8 ± 0.2, and 0.8 ± 0.2, respectively. All resolved rapidly by approximately 90 minutes post-injection (**Fig. 3a, c, and e**). In Cohort B (10 mL), the higher volume of test solution resulted in mean ± SEM Draize scores of 1.4 ± 0.2 (15 min), 0.9 ± 0.3 (15 min), and 0.4 ± 0.2 (30 min) for erythema, swelling, and induration, respectively (**Fig. 3b, d, and f**).

#### HVAI administration

Erythema was minimal for all subjects, with mean ± SEM Draize scores peaking at 1.5 ± 0.1 at 15 minutes post-injection. Erythema resolved for all subjects by 2 h post-injection (**Fig. 4a**).

Minimal injection-site swelling was observed immediately post-injection and remained low for all subjects. The mean ± SEM Draize score for swelling post-injection peaked at 1.0 ± 0.2 at 10 minutes post-injection. By 2 h post-injection, swelling was resolved for 21 subjects; resolution for all 23 subjects was observed by 3 h post-injection (**Fig. 4b**).

The mean ± SEM Draize score for induration post-injection peaked at 0.3 ± 0.1 at 5 minutes post-injection, before gradual resolution (**Fig. 4c**). Induration was barely perceptible for all subjects and resolved by 90 minutes post-injection.

### Injection-site back-leakage

Following syringe pump administration, mean (± SEM) post-injection back-leakage was low and proportional to the injection volume in each cohort: 5.1 ± 1.0 mg for Cohort A (5 mL), and 10.6 ± 1.6 mg for Cohort B (10 mL) (*P* = 0.61). Following HVAI administration, mean (± SEM) post-injection back-leakage was low across both cohorts (8.5 ± 1.9 mg).

These values correspond to back-leakage volumes of 0.005 mL for Cohort A and 0.011 mL for Cohort B following syringe pump administration, and 0.009 mL across both cohorts following HVAI administration, all representing 0.1% of the total injection volume and equivalent to less than 1 drop of liquid.

# References

1. Karcioglu O, et al. A systematic review of the pain scales in adults: which to use? Am J Emerg Med. 2018;36(4):707-14.
